# Welding fume exposure and respiratory innate immune activation: metal-rich particles, inflammatory biomarkers, and immune-informed occupational prevention

**DOI:** 10.3389/fimmu.2026.1882845

**Published:** 2026-08-20

**Authors:** Junjie Wei, Haoyang Ren, Hui Zhang

**Affiliations:** School of Intelligent Engineering, Shandong Management University, Jinan, China

**Keywords:** airway epithelium, host defense, inflammatory biomarkers, macrophage activation, metal-rich particles, respiratory innate immunity, transient receptor potential channels, welding fume

## Abstract

Welding fumes are widely recognized occupational aerosols, yet their effects on respiratory immune pathways remain incompletely integrated into exposure-oriented assessment. Generated by welding materials, process parameters, surface conditions, and ventilation, welding fumes contain fine and ultrafine particles that can deposit in the respiratory tract and interact with epithelial cells, alveolar macrophages, neutrophils, monocytes, and circulating immune cells. This Mini Review examines welding fume exposure as a metal-particle stimulus to the respiratory immune system, focusing on how metal-rich particles initiate oxidative and epithelial–innate immune responses, what inflammatory and host-defense markers reveal about early biological effects, and how such evidence can be interpreted alongside exposure assessment. Evidence from human exposure studies, controlled inhalation studies, cell models, and workplace investigations suggests that welding fumes can induce acute-phase proteins, cytokines, chemokines, and neutrophilic responses, while some studies also indicate altered T helper cell activity and weakened responsiveness to bacterial stimuli. Evidence from related metal and particulate models further suggests that TRPA1/TRPV1-mediated sensory signaling may provide a rapid route to cough and acute airway reflex responses, although direct welding-fume-specific human evidence remains limited. However, interpretation remains constrained by differences in fume composition, particle size, soluble metal fraction, exposure pattern, smoking, infection, co-exposures, and limited longitudinal evidence. These findings support an immune-informed view of welding fume exposure in which inflammatory and host-defense markers help identify biologically active residual exposure. Such markers should not replace exposure limits or engineering controls, but they may strengthen the interpretation of airway immune perturbation in occupational metal-particle exposure.

## 1 Introduction

Welding fume exposure provides a useful occupational setting for examining how metal-rich aerosols perturb respiratory innate immunity before clinically defined disease becomes apparent (1, 2). Beyond their role as airborne contaminants, welding fumes are heterogeneous metal-rich particles that can deposit in the respiratory tract and interact with epithelial cells, alveolar macrophages, neutrophils, monocytes, and circulating immune cells (2–4). This makes welding fume exposure relevant not only to industrial hygiene, but also to respiratory innate immunity, epithelial–immune signaling, and early inflammatory pathway activation (3, 4). Early biological effects may occur before clinically recognizable occupational disease is present.

Several features of welding fume exposure make an immunological reading necessary. The fume itself is chemically complex: depending on the base metal, filler material, flux, coating, and process, it may contain iron, manganese, chromium, nickel, zinc, copper, and other metals in respirable particles (1, 2). In addition, welding generates fine and ultrafine particles that can reach distal airways, interact with epithelial and immune cells, and trigger local and systemic responses (2, 3, 5). The classification of welding fumes as carcinogenic to humans by IARC further reinforces the need to examine biological pathways linking occupational exposure with chronic adverse outcomes, rather than treating exposure limits as the only relevant knowledge base (6, 7).

The immunological question is narrower than the general hazard question. Welding fumes are already recognized as hazardous; the less clearly integrated issue is how metal-rich particles perturb respiratory immune pathways before clinically defined disease becomes apparent. This Mini Review used a targeted narrative search of PubMed, Web of Science, and Scopus through July 2026, combining terms for welding fume, metal particles, respiratory immunity, oxidative stress, biomarkers, host defense, TRPA1, and TRPV1. Priority was given to human occupational, controlled-exposure, and welding-specific mechanistic studies; related metal or particulate models were used to address mechanistic gaps and are identified as indirect evidence. The review focuses on epithelial–innate and sensory-neural signaling, inflammatory and host-defense markers, and interpretation alongside exposure assessment.

## 2 Welding fumes as heterogeneous metal-particle stimuli to the respiratory immune system

Welding fume exposure is highly variable because the hazard is produced by a task system rather than by a single chemical agent. Base metal, electrode or wire composition, surface coatings, shielding gas, welding current, process type, posture, enclosure, and local exhaust ventilation all shape the amount and composition of generated fume (1, 5). This variability matters immunologically because different metals and particle characteristics can trigger different inflammatory pathways (8–10). Mild steel, stainless steel, galvanized steel, and copper- or zinc-containing processes should not be treated as interchangeable immune exposures.

For exposure assessment, this variability has immediate consequences. Conventional exposure assessment often aggregates the task into a measured airborne concentration, such as respirable particulate mass or specific metal concentrations. Such metrics remain essential, but they may not capture all biologically relevant differences. Two tasks with similar mass concentration may differ in particle size distribution, soluble metal fraction, oxidative potential, surface area, and capacity to activate immune cells (5, 8, 9, 11). Conversely, a worker with apparently modest average exposure may experience short high-intensity peaks during confined-space welding, overhead work, poorly positioned local exhaust ventilation, or work on coated materials.

**Figure 1:**
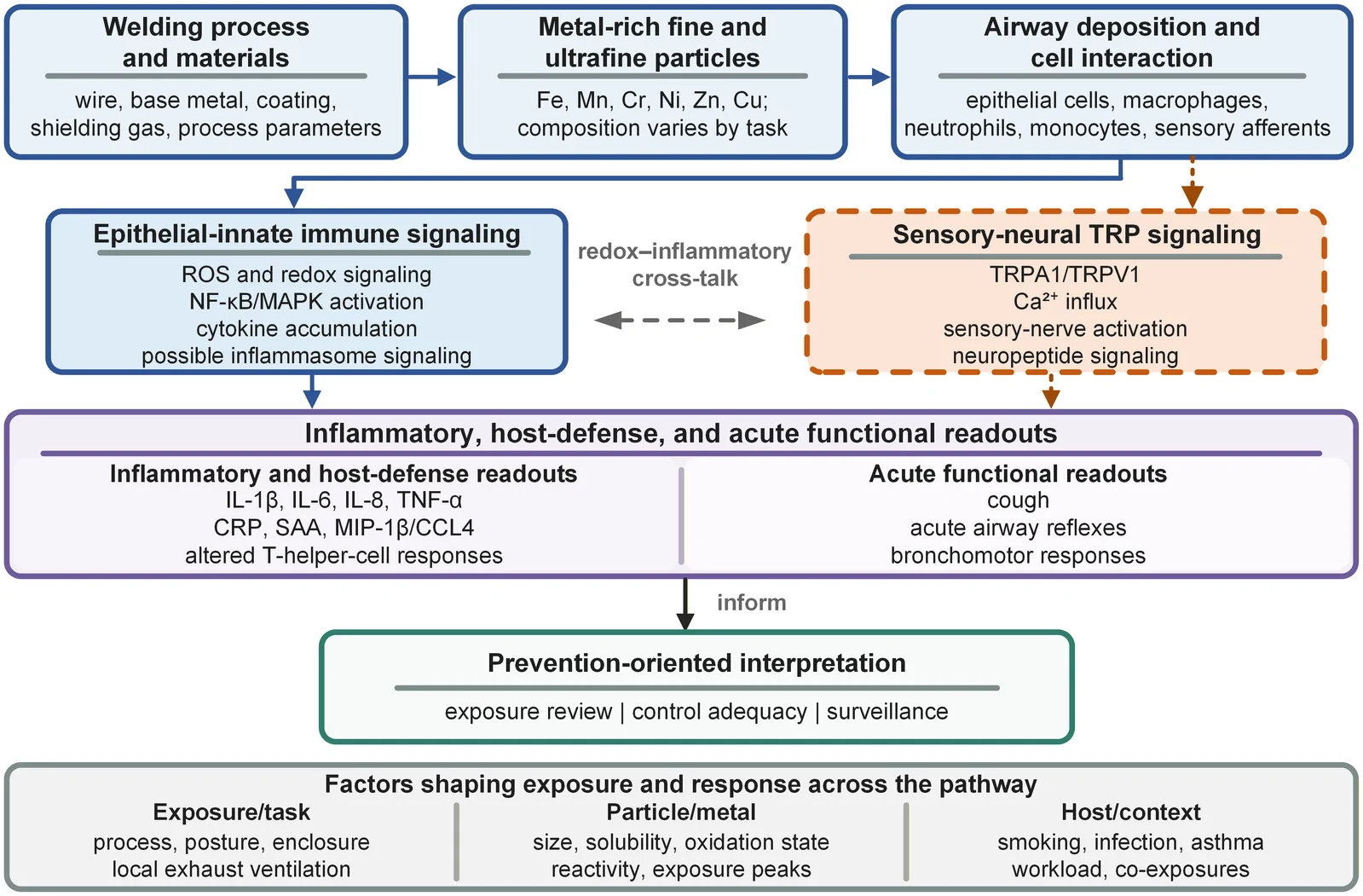
Immune-relevant pathways linking welding fume exposure to respiratory innate immune and sensory-neural activation. Welding processes generate heterogeneous metal-rich fine and ultrafine particles whose effects depend on fume chemistry, particle size, soluble metal fraction, oxidative potential, and exposure pattern. Following airway deposition, particles may interact with epithelial cells, macrophages, neutrophils, monocytes, and airway sensory afferents. Airway deposition may engage parallel epithelial–innate immune and sensory-neural pathways, with potential redox–inflammatory cross-talk between them. TRPA1/TRPV1-mediated Ca²^+^ influx may provide a rapid route to cough and acute airway reflex responses, whereas ROS-driven NF-κB/MAPK signaling supports cytokine accumulation and inflammatory cell recruitment. Direct welding-fume-specific human evidence for the TRP pathway remains limited; it is therefore presented as a mechanistically supported research direction rather than an established welding-fume-specific causal pathway. Solid arrows summarize the principal exposure–response sequence, whereas dashed arrows indicate proposed or indirectly supported TRP-related relationships. ROS, reactive oxygen species; SAA, serum amyloid A; TRP, transient receptor potential.

Welding fume assessment must address both breathing-zone exposure and measurable host responses. Air monitoring remains essential for evaluating contamination and compliance, whereas immune and inflammatory biomarkers indicate whether residual exposure has initiated host responses. Each captures a different part of the exposure-response chain. Biological-effect markers may identify situations in which time-weighted airborne measurements do not capture task variability or residual inflammatory burden. **Figure 1** summarizes these pathways, linking task-generated metal-rich particles to airway deposition, parallel epithelial-innate immune and sensory-neural signaling, inflammatory and host-defense responses, acute functional effects, and prevention-oriented interpretation.

## 3 Metal-particle toxicity, macrophage activation, and epithelial–innate and sensory-neural signaling

The respiratory tract is the primary interface between welding fumes and the immune system. Fine and ultrafine particles can deposit along the conducting airways and alveolar regions, where they encounter epithelial cells, alveolar macrophages, recruited neutrophils, and, indirectly, circulating monocytes and lymphocytes (3, 11–14). Experimental and occupational studies have long suggested that macrophage activation, reactive oxygen species (ROS), and cytokine release are important mechanisms in welding fume-induced lung injury and inflammation (8, 15–17). This mechanism is consistent with a broader particulate-matter paradigm in which particle surface chemistry, soluble metals, redox activity, and oxidative potential influence inflammatory signaling (3, 12, 18–21).

Mechanistically, inhaled metal-rich particles may deposit in the conducting airways or alveolar region, where epithelial cells and macrophages encounter, internalize, or respond to them. Particle surfaces and soluble metal components can promote ROS formation, mitochondrial disturbance, lysosomal stress, and redox-sensitive signaling through NF-κB and MAPK (3, 12, 18). These processes can increase the release of IL-6, IL-8, TNF-α, IL-1β, and chemokines, thereby recruiting and activating leukocytes (16, 17, 22). In some settings, IL-1β release may indicate inflammasome-linked signaling, although current welding-fume studies do not support a single dominant inflammasome pathway across fume types (14, 20, 21).

Alongside these transcriptional and cytokine-centered pathways, redox- and metal-responsive transient receptor potential (TRP) channels may provide a faster sensory-neural route linking metal-rich particles to acute respiratory responses. TRPA1 and TRPV1 are Ca²^+^-permeable channels expressed in airway sensory afferents. Oxidants relevant to particulate exposure can activate TRPA1 (23), whereas selected soluble metal salts have activated TRPV1 in heterologous and primary sensory-neuron models (24). Channel opening promotes Ca²^+^ influx and sensory-neural excitation, potentially contributing to cough, acute airway reflex responses, and neuropeptide-mediated amplification of local inflammation. These electrophysiological events may precede, and subsequently interact with, transcription-dependent cytokine accumulation.

This pathway is mechanistically relevant but not yet welding-specific. Zinc directly activates TRPA1, and zinc and copper stimulate pulmonary sensory neurons through TRPA1 (25, 26). Zinc-, copper-, and iron-containing salts have activated TRPV1 in a non-airway model (24). An ERS Congress abstract reported that inhaled diesel-exhaust particles altered cardiac autonomic balance and enhanced capsaicin-evoked cough in a TRPV1-related human paradigm (27). As this evidence concerns combustion particles and remains a conference abstract, it provides translational context rather than direct proof for welding fumes. Whether welding-fume particles directly engage airway TRPV1 remains unknown. Controlled studies should test whether TRPA1/TRPV1 signaling contributes to acute cough and bronchomotor responses alongside oxidative and cytokine responses.

Macrophages are particularly important because they act both as particle-handling cells and as inflammatory signal amplifiers. A whole-blood assay showed that zinc- and copper-containing welding fume particles increased IL-6, IL-8, TNF-α, and IL-1β after 24 h exposure, indicating an acute systemic inflammatory reaction in a blood immune-cell model (16). More recent work in human alveolar epithelial and macrophage cell lines found that Zn/Cu-containing welding fume particles elicited cell-type-specific responses, with THP-1 macrophage-like cells showing high sensitivity and MIP-1β/CCL4 proposed as a sensitive macrophage activation marker (22). Another study reported that occupationally relevant Zn/Cu-containing metal fumes can inhibit THP-1 macrophage TNF and IL-6 responses to bacterial stimuli, suggesting that welding fume exposure may combine inflammatory activation with altered responsiveness to subsequent microbial challenge (17).

A key interpretive issue is that welding fume exposure may not simply “increase inflammation”. Some evidence suggests a more complex immune phenotype in which inflammatory activation and impaired antimicrobial responsiveness coexist (17, 28, 29). Metal-rich particles can stimulate cytokine and chemokine release under resting conditions, yet repeated or chemically specific exposure may blunt macrophage or T helper cell responses to bacterial antigens (16, 17, 22, 28). This distinction is important because an inflammatory biomarker increase and a reduced response to microbial challenge have different biological meanings. The former indicates activation of innate inflammatory pathways, whereas the latter raises concern about host-defense competence after exposure. Future welding-fume studies should therefore examine basal inflammatory markers and stimulus-induced immune responsiveness together, rather than treating inflammation as a single-direction outcome.

These findings move the discussion beyond nonspecific irritation. Depending on fume chemistry, dose, and timing, exposure may produce a mixed pattern of epithelial injury, macrophage activation, neutrophilic inflammation, cytokine release, and impaired immune competence (8, 10, 16, 17, 22). T helper cell function may also be relevant. In a human study, exposure to welding fumes was associated with suppression of T helper cell activity toward bacterial antigens, raising the possibility that occupational fume exposure can modify host defense as well as induce inflammatory markers (28). Welding fume exposure has also been linked to susceptibility to lower airway infection with Streptococcus pneumoniae (29). Animal and human work on zinc oxide inhalation and metal fume fever further suggests involvement of cytokine networks, including IL-17F, in metal fume-related immune responses (30, 31).

Caution is needed because welding fumes are mixtures rather than standardized immunological stimuli. Stainless steel fumes, mild steel fumes, galvanized material fumes, and copper-zinc fumes may differ substantially in soluble metal fraction, oxidative potential, and immune effect (8–10, 22). Smoking, infection, asthma, existing airway disease, physical workload, and co-exposures can also modify host responses.

Welding fumes should therefore not be treated as a single immunological stimulus. Different fume types may produce different combinations of epithelial stress, macrophage activation, neutrophilic recruitment, cytokine release, and altered antimicrobial responsiveness. Because much recent mechanistic evidence comes from Zn/Cu-containing fumes, extrapolation to stainless-steel, mild-steel, manganese-rich, or mixed-metal fumes requires characterization of particle chemistry, solubility, oxidative potential, and exposure timing (16, 17, 22).

## 4 Inflammatory biomarkers and host-defense markers as immune readouts

Inflammatory biomarkers are immunologically important because they provide readouts of pathway activation before clinically defined disease is evident. In welding fume research, commonly discussed markers include leukocyte and neutrophil counts, C-reactive protein (CRP), serum amyloid A (SAA), IL-6, IL-8, TNF-α, IL-1β, VCAM-1, myeloperoxidase, and chemokines (32–35). These markers do not diagnose a welding-related disease by themselves, but they can indicate that a task or exposure pattern is producing a measurable biological effect. **Table 1** summarizes immune readouts reported in welding fume exposure studies, together with their immunological interpretation, evidence context, interpretive value, and current limitations.

**Table 1:** Immune readouts relevant to welding fume exposure and respiratory immune perturbation.

Endpoint/marker	Immune interpretation	Main evidence context	Interpretive value	Key limitation
CRP	Acute-phase protein induced downstream of inflammatory cytokines	Human exposure and controlled-exposure studies	Candidate signal for identifying tasks associated with systemic inflammatory response	Nonspecific; affected by infection, smoking, obesity, recent illness, and workload
SAA	Acute-phase protein that may respond sensitively to sterile inflammation	Controlled exposure to zinc/copper-containing metal fumes	Complement to CRP in short-term exposure or intervention studies	Clinical meaning for long-term risk remains uncertain
IL-6	Central mediator of acute-phase response	Controlled exposure and workplace biomarker studies	Early biological-effect marker in exposure or control-evaluation studies	Timing of sampling is critical; influenced by many inflammatory conditions
IL-8/CXCL8 and neutrophils	Neutrophil recruitment and airway inflammatory response	*In vitro*, chamber exposure, and human exposure contexts	Candidate indicator of neutrophil-oriented airway inflammation	May not distinguish welding fume effects from irritant or infectious inflammation
TNF-α and IL-1β	Macrophage activation and early pro-inflammatory signaling; IL-1β may indicate inflammasome-linked response	Whole-blood and cell models; selected field studies	Mechanistic indicators for particle-induced immune activation	Not validated as routine occupational surveillance markers
MIP-1β/CCL4	Macrophage-associated chemokine response	Human macrophage-like cell models exposed to Zn/Cu fume	Candidate research marker for macrophage activation	Evidence mainly experimental; field validation needed
T helper cell responses	Adaptive immune responsiveness to microbial antigens	Human immunological study of welding-fume exposure	Potential signal of host-defense alteration, not only inflammation	Requires stronger longitudinal and exposure-characterized evidence

Human evidence supports this early-warning interpretation, while also demonstrating its limits. A study of young welders reported that fine particulate exposure during welding was associated with acute systemic inflammatory responses: PM2.5 concentrations were much higher among welders than controls, CRP increased after exposure, and neutrophil-related responses were observed, with smoking modifying some effects (32). Controlled-exposure studies refined this picture for zinc- and copper-containing fumes. IL-6 has been proposed as an early biomarker for exposure to zinc-based metal fumes (33). SAA has been evaluated as a sensitive marker of subclinical sterile inflammation after inhalation of zinc- and/or copper-containing metal fumes (35). Repeated short-term exposure to zinc- and copper-containing fumes has been associated with persistent increases in systemic inflammatory markers such as CRP (36). Short-term exposure may also induce slight but sustained airway constriction correlated with inflammatory responses (37). The delayed time course—emerging after 24 h and persisting for up to one week—is more consistent with inflammatory or sustained bronchomotor effects than with an immediate TRP-mediated reflex. Future studies should therefore measure cough and airway resistance during and immediately after welding-fume exposure together with TRP-related endpoints.

Other studies broaden the biomarker landscape. Chamber exposure to welding-derived nanoparticle aggregates has been used to evaluate acute respiratory effects and inflammation-related markers under conditions approximating a working day (38), and other studies have examined inflammatory responses during the working day or after welding inhalation challenge tests (39, 40). A metabolomics study reported that welding fume exposure was associated with metabolic changes linked to systemic inflammation (41). A 2025 workplace study further suggested that systemic inflammation may not be confined to welders: in welding factories, office workers as well as welders showed elevated serum concentrations of selected cytokines and chemokines compared with general subjects, highlighting the importance of area segregation, ventilation spread, and protection of adjacent workers (42).

For immune interpretation, the evidence suggests a limited but useful role for biomarker panels. They may reveal exposure patterns that are not captured by a single time-weighted concentration, and panels of markers are likely to be more informative than isolated measurements. Their interpretation, however, remains constrained by nonspecific influences such as smoking, infection, obesity, recent illness, sleep disruption, physical workload, and co-exposure to other airborne contaminants. They are therefore best understood as biological-effect readouts to be interpreted alongside exposure assessment, medical surveillance, and task analysis, rather than as standalone proof of welding-specific harm.

For this reason, no single marker should be interpreted as a welding-specific immune signature. A more defensible approach is to examine marker patterns across compartments and time points, for example combining acute-phase proteins, neutrophil-oriented markers, macrophage-associated chemokines, and stimulus-induced immune responsiveness (17, 22, 32–35). Such panels would help distinguish transient inflammatory activation from persistent immune perturbation or altered host defense.

## 5 Prevention-oriented interpretation of immune evidence

Prevention still depends primarily on exposure reduction through source control, ventilation, task design, and respiratory protection (1, 43, 44). Immune evidence should therefore be used to assess whether residual exposure remains biologically active and to prompt review of control adequacy. This is especially relevant because welding process, material, fume composition, and work setting influence both airborne exposure and immune response.

An immune-informed approach adds a second evaluative layer. Engineering controls are usually judged by reductions in airborne concentration and compliance with relevant limits, and those indicators remain indispensable. However, if workers continue to show consistent inflammatory or host-defense alterations under apparently controlled conditions, this may signal residual exposure peaks, ineffective source capture, air recirculation, inadequate segregation, or unrecognized high-risk tasks. Biomarkers can therefore support control verification when used in a carefully designed surveillance or intervention framework.

This logic is most relevant in work settings where exposure is unstable or poorly bounded, such as construction and maintenance welding, shipbuilding, confined-space repair, work on coated or mixed-metal materials, and workplaces where nearby non-welders may be exposed through air movement or poor segregation (42–44). It is also relevant for evaluating interventions, where biomarker patterns before and after control changes may complement air sampling and task observation.

Biomarker monitoring also requires careful interpretation. Workers should not be treated as biological sensors for inadequate workplace design. Immune markers should be used to prompt exposure-control review, with appropriate medical interpretation, privacy protection, and non-punitive use of results.

## 6 Discussion: unresolved questions and future directions

Available evidence supports a narrower conclusion: welding fumes are complex metal-rich aerosols capable of perturbing respiratory innate immunity and systemic inflammatory signaling. The main controversies no longer concern whether welding fumes can produce biological effects, but how those effects should be explained and used. Three tensions are especially important: whether immune activation is driven mainly by mass concentration or particle reactivity; whether biomarkers represent control-sensitive warning signals or nonspecific inflammatory noise; and whether welding fumes induce only inflammatory activation or also alter antimicrobial host-defense responsiveness.

### 6.1 Mass concentration or particle reactivity: what drives immune activation?

A central unresolved issue is whether immune responses to welding fumes are driven mainly by particulate mass, specific metal constituents, soluble metal fractions, particle number, surface area, oxidative potential, or short peak exposures. This distinction matters because occupational exposure assessment often relies on time-weighted airborne concentration, whereas immune activation may be shaped by properties that are not fully captured by mass-based metrics. Two welding tasks with similar measured fume mass may differ substantially in particle size distribution, metal solubility, redox activity, and capacity to activate epithelial cells, macrophages, or neutrophils (5, 8–10, 15). Future studies should therefore combine personal exposure monitoring with physicochemical characterization of particles and immune endpoints. Such designs would help distinguish a general aerosol burden from metal-specific or particle-reactivity mechanisms. An additional priority is to determine whether particle reactivity is translated into acute respiratory symptoms through TRPA1/TRPV1-dependent sensory signaling. Controlled-exposure studies combining fume composition, oxidative potential, cough-reflex testing, airway resistance, autonomic measures, and TRP-related endpoints could distinguish rapid sensory-neural responses from delayed inflammatory changes (23–27).

### 6.2 Are inflammatory biomarkers immune-relevant warning signals or nonspecific inflammatory noise?

Inflammatory biomarkers occupy an ambiguous position in the immune interpretation of occupational particle exposure. Acute-phase proteins such as CRP and SAA may reflect systemic inflammatory responses after welding-related metal fume exposure (32, 33, 35). Cytokines and chemokines, including IL-6, IL-8, TNF-α, IL-1β, and CCL4/MIP-1β, provide more direct evidence of innate immune activation in controlled exposure, whole-blood, and cell-model studies (34, 36–40). These markers are useful as early biological-effect signals, but their interpretation remains constrained by nonspecific influences such as smoking, infection, obesity, recent illness, physical workload, sleep disruption, and co-exposures. Their value lies less in diagnosing welding-related disease or replacing occupational exposure limits than in indicating that residual exposure may be producing measurable biological effects. A more defensible use is to treat them as control-sensitive warning signals: evidence that residual exposure may be producing biological effects even when conventional air measurements appear acceptable. Longitudinal studies are still needed to determine whether short-term inflammatory responses predict persistent inflammation, lung-function change, infection susceptibility, occupational asthma, cardiovascular risk, or cancer-relevant pathways.

### 6.3 Does welding fume exposure activate inflammation while weakening host defense?

A further unresolved question is whether welding fume exposure produces a dual immune effect: inflammatory activation under baseline conditions and reduced responsiveness to microbial challenge. Evidence of altered T helper cell activity, impaired macrophage cytokine responses to bacterial stimuli, and increased susceptibility to lower airway infection suggests that inflammation alone may not capture the full immune consequence of exposure (17, 28, 29). Future studies should therefore include challenge-based immune assays, infection-related endpoints, and longitudinal follow-up, rather than relying only on resting cytokine or acute-phase protein levels.

## 7 Conclusion

Welding fume exposure provides a useful occupational model of metal-particle-induced immune perturbation. Current evidence indicates that metal-rich fumes can activate epithelial–innate immune signaling, macrophage and neutrophil responses, cytokine and chemokine release, acute-phase pathways, and, in some settings, altered host-defense responsiveness. The main challenge is not simply to show that inflammation occurs, but to identify which particle properties, exposure patterns, and susceptible host conditions determine the immune phenotype. TRPA1/TRPV1-mediated sensory signaling may provide a parallel, rapid route to cough and acute airway reflex responses, but direct welding-fume-specific human evidence remains limited. Inflammatory and host-defense markers should therefore be interpreted as biological-effect signals that complement, rather than replace, exposure assessment and preventive control.
